# A genome epidemiological study of mycobacterium tuberculosis in subpopulations with high and low incidence rate in Guangxi, South China

**DOI:** 10.1186/s12879-021-06385-0

**Published:** 2021-08-19

**Authors:** Dingwen Lin, Junning Wang, Zhezhe Cui, Jing Ou, Liwen Huang, Ya Wang

**Affiliations:** 1 Department of Nutrition and School Health, Guangxi Zhuang Autonomous Region Center for Disease Control and Prevention, Nanning, China; 2 Zeta Biosciences(Shanghai) Co.,Ltd., Shanghai, China; 3 Department of Tuberculosis Control, Guangxi Zhuang Autonomous Region Center for Disease Control and Prevention, Nanning, China

**Keywords:** Molecular epidemiology, Mycobacterium tuberculosis, China, Drug resistance, Genetic evolution

## Abstract

**Background:**

Tuberculosis (TB) is caused by a bacterium called *Mycobacterium tuberculosis* (Mtb). China is the third in top 8 high TB burden countries and Guangxi is one of the high incidence areas in South China. Determine bacterial factors that affected TB incidence rate is a step toward Ending the TB epidemic.

**Results:**

Genomes of *M. tuberculosis* cultures from a relatively high and low incidence region in Guangxi have been sequenced. 347 of 358(96.9%) were identified as *M. tuberculosis*. All the strains belong to Lineage 2 and Lineage 4, except for one in Lineage 1. We found that the genetic structure of the *M. tuberculosis* population in each county varies enormously. Low incidence rate regions have a lower prevalence of Beijing genotypes than other regions. Four isolates which harbored *mutT4*-48 also had *mutT2*-58 mutations. It is suggested that strains from the ancestors of modern Beijing lineage is circulating in Guangxi. Strains of modern Beijing lineage (OR=2.04) were more likely to acquire drug resistances than Lineage 4. Most of the lineage differentiation SNPs are related to cell wall biosynthetic pathways.

**Conclusions:**

These results provided a higher resolution to better understand the history of transmission of *M. tuberculosis* from/to South China. And the incidence rate of tuberculosis might be affected by bacterial population structure shaped by demographic history. Our findings also support the hypothesis that Modern Beijing lineage originated in South China.

## Background

**Fig. 1:**
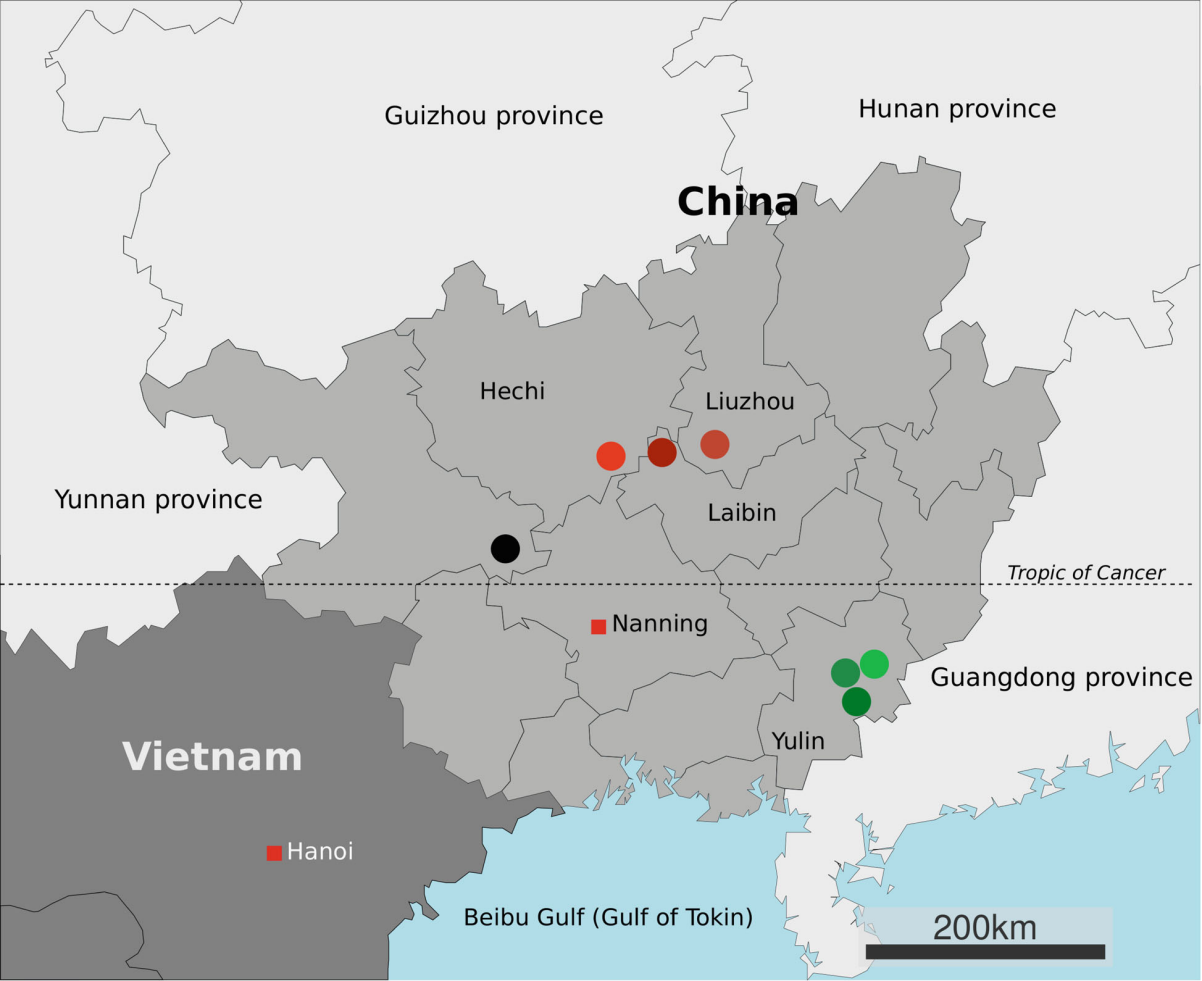
Approximate geographic locations of *M. tuberculosis* strains sampling sites in Guangxi, China. The fill color of provincial administrative regions of China next to Guangxi is light grey, the color of foreign countries is dark grey. The capitals of countries or provinces have marked by a red square. Red and brown round dots denote the counties in hot spot, green dots indicate cold-spot. Hot spot is a term in geology that referred to *M. tuberculosis* high incidence rate area and vice versa. The black dot is a county that did not identify as a hot or cold spot in our previous study

Tuberculosis (TB) is caused by *Mycobacterium tuberculosis* that most often affect the lungs. Top eight high TB burden countries accounted for two thirds of new TB cases globally in 2019. One of the milestones of the WHO Ending TB Strategy is new TB cases drop 80% by 2030. China is the third in top 8 high TB burden countries and the Guangxi Zhuang Autonomous Region is one of the high incidence provincial administrative region in South China. In our previous studies, we identified hot spots (high TB incidence rate areas) and cold spots(low TB incidence rate areas) in Guangxi by epidemiological methods and spatiotemporal scanning technology [1]. It is must be said that national and provincial assistance for a TB high incidence region (217.2 per 100,000 population) in north-central Guangxi and a low TB incidence rate region (31.2 per 100,000 population) in south-eastern Guangxi is almost the same (Fig. 1). In this context, determine bacterial factors that affected TB incidence rate is a way to optimize our investments in ending the TB epidemic. We have investigated some of the environmental factors in previous research. In this study, we hypothesize that the pathogenicity of *M. tuberculosis* strains in the hot and cold spot is different. we try to characterize the genetic background of the pathogen and figure out some potential associating factors.

## Result

### Epidemiological characteristics of study participants

The people in the cold-spot mainly speak Cantonese as their first language. In the hot spots south-western Mandarin is generally adopted. Language differences reflect the distinct demographic histories of TB human hosts. Genome sequences of 358 bacterial cultures have been determined with WGS. The minimal sequencing depth of each sample is 100 × of H37Rv genome size. Eleven (11/358 [3.0%]) patient samples were identified as Nontuberculous Mycobacteria (NTM) or *Mycobacterium tuberculosis* contaminated with other respiratory pathogens. The remaining 357 samples were identified as *M. tuberculosis* strains. One from central Guangxi was identified as Lineage1.1.1.1(L1.1.1.1). 215 samples were recognized as L2 (East Asia) and 130 samples were recognized as L4 (Euro-American).

### Cluster analysis by genome-wide SNPs

**Fig. 2:**
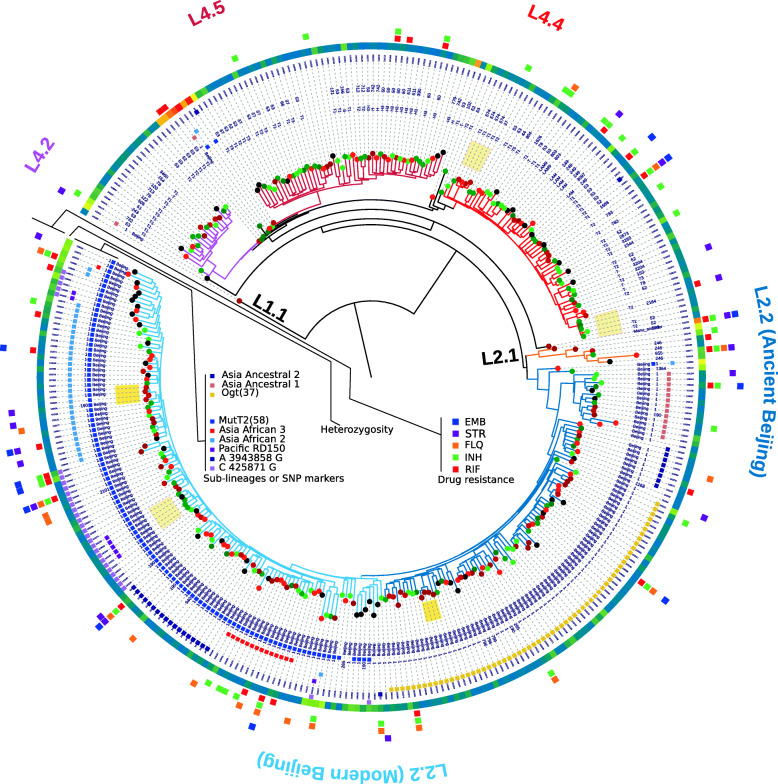
Genetic profiling of 347 *M. tuberculosis* strains from Guangxi, China. Phylogenetic tree reconstructed by maximum-likelihood algorithm using 26870 single-nucleotide polymorphism sites. Heatmap employing a cool-to-warm (value from 0 to 1) color scheme to represent the heterozygosity of every haploid genome. Heterozygosity of problematic leaf-nodes that have branch length and topological variation is high. Outer circles of heatmap marked drug resistance detected by in silico method. Lineage-specific genetic markers labeled in the inner circles used color squares. Dots that denote the leaf-nodes are corresponding to color dots in Fig. 1. And potential local circling clusters are highlighted in yellow

**Fig. 3:**
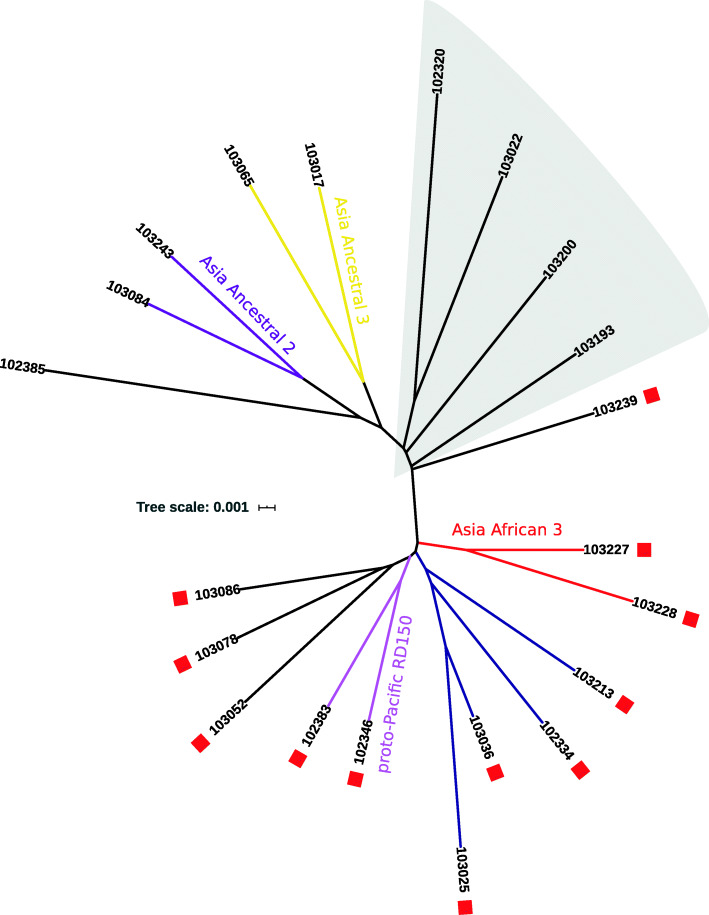
Maximum-like Phylogeny of proto-Modern Beijing strains from Guangxi, China. Shows the evolutionary position of several proto-Modern Beijing strains. Branch color is corresponding to Fig. 2. Red squares beside the leaf-nodes indicates this sample has lineage specific *ogt*-12 SNV

Maximum likelihood phylogeny was used to determine the population structure of 347 *M. tuberculosis* cultures. A single DR-TB case of L1.1.1.1 was detected in central Guangxi which was considered an endemic lineage in Vietnam only [2]. *M. tuberculosis* L2 in the Guangxi area is composed of L2.1(proto-Beijing) and L2.2(modern- and ancient-Beijing sub-lineage). Euro-American lineage consists of sub-lineages L4.2, L4.4, and L4.5. Some of the *M. tuberculosis* cultures appeared to be mixed infections (indicated by lineage specific SNPs, genotype heterozygosity and distance from leaf node to lineage common ancestor). At the very least, 4 Modern Beijing strains in L2.2 mixed with L4 or ancient Beijing lineage can be identified (Fig. 2). Ancestral Beijing strains in this study consist of Asia ancestral 1, 2 and 3. There are four sub-lineages of known classification in Modern Beijing strains, Asian African 1, 2, 3 and Pacific RD150. Only a single sample belongs to the Asian African 1 group. According to our study, over 30 strains assigned to the Modern Beijing lineage (harbored *mutT2*-58 mutations) cannot be subdivided into unified classifications [3]. We have developed two SNP markers to describe these strains. One SNP is 3943858 A/G which Pacific RD150 strains shared, the other one is 425871 C/G (Fig. 2). Interestingly, several strains are close to the ancient Beijing genotype and are considered as Modern Beijing lineage without *mutT2*-58 mutations. All these genomes share the mutation 3048912 C/G which is a common ancestral genetic marker of Modern Beijing lineage and group Bmyc26. The phylogeny shows that these *M. tuberculosis* strains’ evolutionary position is between recognized Modern and Ancient Beijing sub-lineages. Both *mutT2*-58(1286766 G/C, codon 58) and *ogt*-12 (1477596 C/T, codon 12) mutations cannot be detected in these strains, except sample 103239 which has an *ogt*-12 SNV (Fig. 3). It is almost impossible to obtain perfect data in clinical cultures [4]. Cross-contamination and/or mixed infection of multiple lineages of tuberculosis can affect the topology of the tree by Pseudo-homoplasy phylogenetic signal. To help with data interpretation, we used the heterozygosity of SNPs to estimate and label the problematic leafs.

### *M. tuberculosis* genetic differences and lineages expansion in geographic spaces

The genetic population structure of *M. tuberculosis* in high and low incidence rate areas in Guangxi is quite different. The most prevalent *M. tuberculosis* lineage in this study is L2 which contributed 62% (215/346) of TB cases. On the other hand, the proportion of L4 in the low burden region is 47.8% (68/142). Our result shows that almost half of the patients in the southeast of Guangxi infected by strains in Europe-originated lineage. This result is quite different from the high incidence rate area of Guangxi which has a L4 proportion of 30.6% (45/147) and previous surveillance in China [5]. We combined the geographic areas (administrative area with several counties) of *M. tuberculosis* cultures origin and phylogeny. Two monophyletic groups with geographic links were observed which suggest that clonal expansion and circulation of Beijing lineage occurred within these counties. And three clusters are endemic in hot spot or cold spot regions only (Fig. 2). There is no recent transmission event that can be observed in this study by using a cgMLST scheme consisting of 2891 core *M. tuberculosis* gene [6] with 12 SNPs distance cut-off [7], also see (Fig. 2).

### Antimicrobial resistance and pathogenesis of *M. tuberculosis* L2/L4

Modern Beijing lineage of *M. tuberculosis* exhibits more drug resistance than L4 strains. But the Ancient Beijing lineage has similar rate of susceptibility with L4. Compared to L4 strains endemic to South China, over 25.89% (29/112) of the Modern Beijing strains harbored drug-resistant mutations. This rate is statistically and significantly higher than the Ancient Beijing lineage of 14.58% (14/96). We have counted the genetic mutations of anti-tuberculosis drug resistance. The drug-resistant mutations of Ancient Beijing lineage is similar to L4 in Guangxi. *M. tuberculosis* Modern Beijing lineage has a significant increase of drug-resistant mutations than *M. tuberculosis* Lineage 4. The number of resistant mutations in Modern Beijing strains is about two times that of L4 or Ancient Beijing lineage (Tables 1 and 2).

**Table 1:** In-silico drug-resistance rate of dominated *M. tuberculosis* lineages, Guangxi, China, 2018^1^

*M. tuberculosis* Lineage	DR Isolates, no.(%)	Odds ratio (95% CI)	*P*-value
L4	19/130(14.61)	-	-
L2.2A^2^	14/96(14.58)	0.997(0.435 - 2.238)	1.000
L2.2M^3^	29/112(25.89)	2.035(1.023 - 4.127)	0.035

^1^Resistant to at least one anti-TB drug. Fisher’s Exact Test conducted in R.

^2^Ancestral Beijing lineage in Lineage 2

^3^Modern Beijing lineage in Lineage 2

**Table 2:** Number of drug-resistance mutation by *M. tuberculosis* lineages, Guangxi, China, 2018^1^

*M. tuberculosis* Lineage	Isolates no.	DR Mutations no.	Odds ratio (95% CI)	P-value
L4	130	31	-	-
L2.2A^2^	96	23	1.004(0.523 - 1.906)	1.000
L2.2M^3^	112	51	1.905(1.110 - 3.309)	0.015

^1^Number of drug-resistance mutations across all the strains. Fisher’s Exact Test conducted in R.

^2^Ancestral Beijing lineage in Lineage 2

^3^Modern Beijing lineage in Lineage 2

### NTM and mixed infection of *M. tuberculosis*/NTM

Eleven samples were considered contaminated. We used ANIm and AF metrics [8] to identify these genomes. ANIm greater than 0.95 (or known as sequence similarity of 95%) and aligned fragment length (AF) over 60% of the reference genome were considered as the same bacterial species with reference. We have confirmed that 5 of 359 samples were clinically identified as *M. tuberculosis* but was actually NTM. And the rest of the samples were contaminated with bacteria from the respiratory tract and oral cavity Table 3 shows a list of NTM and mixed infection of *M. tuberculosis*/NTM.

**Table 3:** Genome binning and identification of NTM and mixed infection of *M. tuberculosis*/NTM from Guangxi, China, 2018

Sample	Reference Assembly	Reference genome name	ANIm	AF
102031	GCA_001403655.1	*Mycolicibacterium peregrinum*	0.9831	0.8634
102154	GCA_002086345.1	*Mycobacterium marseillense*	0.9936	0.9202
102332	GCA_000277125.1	*Mycobacterium intracellulare*	0.9901	0.9346
103004	GCA_000195955.2	*Mycobacterium tuberculosis*	0.9994	0.9908
103008	GCA_000353205.1	*Mycobacterium orygis*	0.9986	0.9947
103020	GCA_001186365.1	*Gordonia jacobaea*	0.9646	0.3282
103020	GCA_002454895.1	*Gordonia sp.* UBA6683	0.9717	0.5990
103057	GCA_002105755.1	*Mycobacterium colombiense*	0.9950	0.9739
103138	GCA_002024265.1	*Bacillus flexus*	0.9913	0.8317
103204	GCA_001810825.1	*Streptococcus sp.* HMSC076C08	0.9504	0.8394
103204	GCA_000195835.2	*Mycobacterium tuberculosis*	0.9987	0.9927
103209	GCA_001071995.1	*Streptococcus sp.* 263_SSPC	0.9453	0.8155
103209	GCA_000195835.2	*Mycobacterium tuberculosis*	0.9987	0.9931
103237	GCA_000478175.1	*Corynebacterium sp.* KPL1814	0.9421	0.8311
103237	GCA_000195955.2	*Mycobacterium tuberculosis*	0.9990	0.9931

## Discussion

We conducted a preliminary survey in Guangxi using the genome epidemiology method. Except for one strain of Lineage1.1.1.1 which originated from Vietnam, *Mycobacterium tuberculosis* in Guangxi belonged predominantly to sub-lineages L2.1(proto-Beijing), L2.2(Modern- and Ancient- Beijing), L4.2, L4.4 and L4.5. This result is similar to previous nation-wide genomic studies [5].

The bacterial population structure in Yulin is different from inland China even the hot spot in central-west Guangxi. Almost half of the strains in Yulin(cold spot) belong to *M. tuberculosis* L4 which originated in Europe [9]. A large number of migrants from south-east Guangxi moved to the Southeast Asian colonies of western powers, for example as economic migrants or victims of human traffickers, since the Ming dynasty (1368 - 1644 AD) this continued up to as recently as the 1950’s.Some of the Chinese laborers returned to their hometown in different phases of history. These Chinese expatriates might be carriers of *M. tuberculosis* L4 strains of Southeast Asia.

Mainly, the Ancient Beijing lineage in Guangxi consists of three sub-lineages including the Asian Ancestral 1, 2 and 3. And the Modern Beijing lineage in Guangxi consists of Asia Africa 2, Asia Africa 3 and two sub-lineages which is unclassified in the unified schema [3]. A few strains without sub-lineage specific markers might be attached to the root of sub-trees. Sub-trees of two unnamed Modern Beijing sub-lineages can be defined using SNPs 425871 C/G and 3943858 A/G as a specific marker, respectively. Pacific RD150 is a sub-group of the clade of 425871 C/G which is observed in Yulin only and that is specific for the Pacific region [10]. We also found some strains of intermediate genotype between the Modern and Ancient Beijing genotype (Fig. 2). These findings support a hypothesis that the Modern Beijing lineage of *M. tuberculosis* originated in South China [11].

Beijing genotypes could be considered as ‘native strains’ and dominated in mainland China, hence Lineage 4 of *M. tuberculosis* is an ‘exotic species’. Even though the transmission and pathogenesis of *M. tuberculosis* strains in Modern Beijing lineage are considered higher than L4 strains, the prevalence of *M. tuberculosis* L4 is almost the same as the Beijing genotype in the communities of returned Chinese expatriates in South China. Theoretically, L2 and L4 have the same ecological niche. *M. tuberculosis* strains in L4 might have stronger colonization ability in the newborn’s respiratory tract and it might be transmitted from close relatives.

Previous study has inferred that *M. tuberculosis* genes in cell wall envelop bio-genesis under strong diversifying selection and might be related to *M. tuberculosis* lineages specialization [12]. Our population study revealed that lineages differentiation of *M. tuberculosis* L2 and L4 in the molecular level are cell wall bio-synthesis related genes (Table 4). In other words, the composition of mycobacterial cell envelope or cell wall is a big biological difference between *M. tuberculosis* lineages. For instance, one of the SNPs we have observed in *accD4* gene between L2 and L4 is 4254431 C/T. *M.tuberculosis* contains six ACCase (AccD1-6). Previous studies indicate that AccD4, AccD5, and AccD6 are important for cell envelope lipid biosynthesis and that its disruption leads to pathogen death. A synonymous mutation will not change the structure of the protein but would affect the amount of *accD4* protein at the transcriptional level [16]. AccD4 was proposed to form Acyl-CoA carboxylases complex with AccD5 and accept propionyl-CoA as a substrate to produce methylmalonyl-CoA, which is the building block of mycocerosic acid [17].

**Table 4:** Estimation of SNPs that might have been subject to stabilizing or diversifying selection between *M. tuberculosis* Lineage 2 and 4, Guangxi, China, 2018

SNP Position	Gene	*F*_*ST*_	RefCodon	CodonPos	AlterCodon	AA variation	Subcellular location	CDS Product Annotation
1230778	*mazF3*	0.9703	ACC	65	ATC	T/I	unknown	Toxic component of a type II toxin-antitoxin (TA) system.
								It dramatically increases persister cell formation
								in M.smegmatis upon challenge with gentamicin or kanamycin.
								Probable propionyl-CoA carboxylase beta chain 4 AccD4.
4254431	*accD4*	0.9644	GAC	506	GAT	D/D	Cell wall	mycolate cell wall layer assembly.
3408150	*Rv3047c*	0.9644	ACA	53	GCA	T/A	unknown	Hypothetical unknown protein.
3027798	*Rv2714*	0.9644	GTG	245	GCG	V/A	plasma membrane	Conserved alanine and leucine rich protein.
								Identified in the membrane fraction of *M. tuberculosis* H37Rv
								confers non-target based resistance to azoles,
776100	*mmpL5*	0.9585	ACC	794	ATC	T/I	integral component of plasma membrane	clofazimine and bedaquiline,
								via an efflux mechanism.
738522	*mmaA2*	0.9585	GAA	213	GAC	E/D	plasma membrane	Involved in mycolic acids modification.
3530955	*Rv3161c*	0.9585	GTC	62	CTC	V/L	plasma membrane	Possible dioxygenase
3266030	*ppsD*	0.9585	TCA	1261	TCG	S/S	plasma membrane	Phenolpthiocerol synthesis type-I polyketide synthase PpsD
262268	*Rv0219*	0.9585	GCA	5	GCT	A/A	Cell wall	Probable conserved transmembrane
								Probable dihydroorotate dehydrogenase PyrD.
2399734	*pyrD*	0.9585	GGC	339	AGC	G/S	plasma membrane	Essential gene for in vitro growth of H37Rv,
								by analysis of saturated Himar1 transposon libraries [13]
931123	*lpqQ*	0.9527	TAT	57	TAC	Y/Y	extracellular region	Possible lipoprotein LpqQ
								ESX conserved component EccA3.
342146	*eccA3*	0.9527	GAA	6	GCA	E/A	plasma membrane	ESX-3 type VII secretion system protein.
								Part of the ESX-3 specialized secretion system.
2388641	*ansP1*	0.9527	GGC	9	GAC	G/D	integral component of membrane	L-asparagine permease AnsP1
2138453	*Rv1888A*	0.9527	GGG	55	GGA	G/G	Cell wall	Conserved hypothetical protein
213147	*sigG*	0.9527	GAC	332	TAC	D/Y	unknown	Probable alternative RNA polymerase sigma factor SigG

Genome coordinates correspond to reference genome sequence AL123456.3. Subcellular location and CDS product annotation based-on UniProt [14] and TubercuList [15].

The role of the cell wall of *M. tuberculosis* has been involved in pathogenesis [18]. Molecular structural diversity on the surface of bacteria may affect interaction with human epithelial cells or macrophages. Another role of *M. tuberculosis* cell wall is conferring to resistance to many anti-tuberculosis agents.

Some of the previous studies inferred that the Beijing genotype of *M. tuberculosis* is less associated with drug resistance in South China [19, 20]. Meanwhile, surveillance overseas purposed that the Beijing genotype of *M. tuberculosis* is a risk factor for drug resistance [21]. Interestingly, our calculation suggested that strains in the Modern Beijing sub-lineage have more drug resistance mutations than Lineage4 in South China other than strains of the Ancient Beijing genotype. Conflicts of previous studies might be due to the ignorance of genetic segregation. Clonal expansion is a common phenomenon in *M. tuberculosis* transmission. In our preliminary surveillance, we observed several clusters circulating in counties or prefecture cities of Guangxi. From a public health aspect, although financial and technical support for counties in the hot spot and cold spot from provincial CDC are almost the same, TB burden is quite different. Our study purposed that the related high proportion of Beijing lineage(L2) in hot spot area might be one reason for the higher incidence rate.

Another clinically relevant issue is the misdiagnosis of Nontuberculous Mycobacteria (NTM) infections. About 3% [11/358] of the culture-based laboratory *M. tuberculosis* diagnostics is wrong. The probable causes of misdiagnosis might be mixed infection/contamination and similarity of culture-based phenotypes determination.

In recent years, WGS-based approaches are more and more applied in *M. tuberculosis* research, especially in surveillance projects. It has become the standard technology for *M. tuberculosis* molecular epidemiology and the application in clinical settings is gaining acceptance (32). One of the considerations of infectious disease surveillance is the detection of recent transmission events [22]. There is no unified clustering method for genome-wide SNPs based typing yet [22]. In this work, we did not find any recent transmission event in this study by using a cgMLST scheme consisting of 2891 core *M. tuberculosis* genes and 20 SNPs cut-off [7]. The small sample size might be the reason for the failure to detect recent transmission clusters. The topic of recent transmission of *M. tuberculosis* in Guangxi should be investigated in further studies.

Another problem in whole-genome wide SNPs-based phylogenic analysis of *M. tuberculosis* is laboratory contamination and/or a mixture of *M. tuberculosis* lineages in clinical samples. For rapid outbreak tracking, public health laboratories often direct use materials submitted by the health care provider. These materials might be patient specimens or initial growth in Broth which would submit to the sequencing process without further isolation of bacteria strains. As a result, contaminations in the sequencing data can not be avoided. When the sample size is increased, the number of shared homogeneous SNP sites across the samples is decreased. If heterozygous SNPs are used, some conflicting phylogenetic signals would affect the phylogenetic tree, and pseudo-homoplastic clades would be introduced. These clades make data interpretation more difficult. To facilitate data interpretation, we used heterozygosity as a metric to indicate the problematic leaf-nodes in the tree, which leads to a more robust and solid conclusion. We noticed that some recent studies [23, 24] mentioned that they used heterozygous sites as high-confidence variations to perform tree construction. That may be a neglected problem in research laboratories because alternate data-set is available. It should be considered that the structure of the phylogenetic tree could be influenced by heterozygous sites.

The rate of resistance acquisition is determined by several factors including the population size, mutation rate and mutational target size [25]. L2 has been associated with greater drug resistance compared to L4 and the other ancient lineages [25]. The influence of strain’s genetic background on the rate of resistance acquisition has been much debated. Our results suggested that Modern-Beijing strains have greater antibiotic-resistance mutations than Ancient-Bejing strains and strains in L4. Theoretically, there are two possible explanations, one is the rate of resistance acquisition of Modern-Beijing lineage higher than others, another one is bacteria under the selection pressure of drugs and expanded via patient-to-patient transmission. We have no determine contribution of each of these two factors. But to prevent drug-resistant TB develop during treatment, it is reasonable that a high-resolution genotype of *M. tuberculosis* should be considered in the clinical setting when it is available.

## Conclusions

We provided a higher resolution to better understand the history of the transmission of *Mycobacterium tuberculosis* in South China. Genetic structures of *M. tuberculosis* is different in hot and cold spot. Genetic background of *M. tuberculosis* may influence the TB burden. Geographical pathogen population structure which is shaped by demographic history and local transmission events should be a concern in public health policymaking.

## Methods

### Case inclusion and epidemiology data collection

We conducted a retrospective study to identify the bacterial genetic factors affecting TB incidence.The sample size of this study was determined based on a previous epidemiological survey. Patients above 5-years-old and diagnosed with pulmonary tuberculosis between January to June in 2018 from hot and cold spots were down-sampled. All the data entries of selected patients with culture confirmed pulmonary tuberculosis were retrieved from the National Notifiable Disease Reported System of China by a criterion as follows: cultured positive *M. tuberculosis* isolates from sputum samples corresponding to patients.

### DNA extraction and WGS

DNA of positive *M. tuberculosis* culture isolates were extracted using a genetic sample kit (HiPure Bacterial DNA Kit, Magen Biotech Co. Ltd). 150-bp paired-end shotgun whole-genome sequencing (WGS) of 359 isolates was performed on the Illumina (San Diego, California) NovaSeq platform using the Nextera XT DNA sample preparation kit according to the manufacturer’s instructions.

### Genome sequences analysis

*M. tuberculosis* WGS data files in the FASTQ format (2 ×150-bp reads) of each sample were trimmed with Trimmomatic [26] v0.3.2. Trimmomatic cut bases off the start of a read if quality score is under 30 and retain reads at a minimal length of 91 bp. Trim paired-end reads were aligned using Bowtie2 [27] v2.3.0 against the genome of *M. tuberculosis* H37Rv (Genbank accession: AL123456.3) with the parameter “–very-sensitive”. SNPs were identified using VarScan [28] v2.3.8 and SAMtools [29] v1.3.1, variations with coverage under 30 × or the minimal frequency of homozygote below 0.99 were ignored. Further filter was applied to all the VCF files using bcf tools v1.9. In our filter criteria, heterozygous SNP or SNP within 5 bp of an indel was filtered. SNPs with genotype quality below 255 or below a minimum of 90% of the median coverage in each bam file were discarded [30]. Variations in x the coding sequence of proteins that contain PE or PPE motifs [31] were characterized by Genebank’s annotation and known drug resistance genes were discarded. Anti-TB drug resistance SNPs were identified using TB-Profiler [32] and an additional database which was in-house curated. Finally, 26869 SNPs remained after filters. VCF-kit [33] was used to concentrate all the remaining SNPs into pseudo-sequences for phylogeny analysis. All of these SNPs were loaded into a MongoDB database and performed queries using Perl script with MongoDB driver.

A maximum-likelihood phylogeny was reconstructed using RAxML [34] v8.1.9 with a general time reversible (GTR) nucleotide substitution model and 100 bootstrap replicates. De novo assembly of reads used SPAdes [35] v3.13.0, and the binning used MyCC.py [36]. Bacterial species of binned contigs were identified by ANIm with a cut-off 0.95 using in-house scripts. All statistical analyses were performed in R-language v3.4.4 unless otherwise stated. *M. tuberculosis* lineages were assigned and Beijing strains sub-lineages used Coll’s 62 SNPs schema [37]. In silico spoligotyping used SpoTyping-v2.0 [38]. Data visualization used the online tool iTOL [39].

## Data Availability

The gene sequences have been deposited in the Genome Sequence Archive in Beijing Institute of Genomics (BIG) Data Center, Chinese Academy of Sciences, under accession numbers PRJCA002021 and are publicly accessible at https://bigd.big.ac.cn/gsa.

## References

[1] Cui Z, Lin D, Chongsuvivatwong V, Zhao J, Lin M, Ou J, Zhao J (2019). Spatiotemporal patterns and ecological factors of tuberculosis notification: A spatial panel data analysis in Guangxi, China. PLoS ONE.

[2] Holt KE, McAdam P, Thai PVK, Thuong NTT, Ha DTM, Lan NN, Lan NH, Nhu NTQ, Hai HT, Ha VTN, Thwaites G, Edwards DJ, Nath AP, Pham K, Ascher DB, Farrar J, Khor CC, Teo YY, Inouye M, Caws M, Dunstan SJ (2018). Frequent transmission of the Mycobacterium tuberculosis Beijing lineage and positive selection for the EsxW Beijing variant in Vietnam. Nat Genet.

[3] Shitikov E, Kolchenko S, Mokrousov I, Bespyatykh J, Ischenko D, Ilina E, Govorun V (2017). Evolutionary pathway analysis and unified classification of east asian lineage of mycobacterium tuberculosis. Sci Rep.

[4] Wyllie DH, Robinson ER, Peto T, Crook DW, Ajileye A, Rathod P, Allen R, Jarrett L, Smith EG, Walker AS, Forbes BA (2018). Identifying Mixed Mycobacterium tuberculosis Infection and Laboratory Cross-Contamination during Mycobacterial Sequencing Programs. J Clin Microbiol.

[5] Liu Q, Ma A, Wei L, Pang Y, Wu B, Luo T, Zhou Y, Zheng H-X, Jiang Q, Gan M, Zuo T, Liu M, Yang C, Jin L, Comas I, Gagneux S, Zhao Y, Pepperell CS, Gao Q (2018). China’s tuberculosis epidemic stems from historical expansion of four strains of mycobacterium tuberculosis. Nat Ecol Evol.

[6] Kohl TA, Harmsen D, Rothgänger J, Walker T, Diel R, Niemann S (2018). Harmonized genome wide typing of tubercle bacilli using a web-based gene-by-gene nomenclature system. EBioMedicine.

[7] Meehan CJ, Moris P, Kohl TA, Pečerska J, Akter S, Merker M, Utpatel C, Beckert P, Gehre F, Lempens P, Stadler T, Kaswa MK, Kühnert D, Niemann S, de Jong BC (2018). The relationship between transmission time and clustering methods in mycobacterium tuberculosis epidemiology. EBioMedicine.

[8] Varghese NJ, Mukherjee S, Ivanova N, Konstantinidis KT, Mavrommatis K, Kyrpides NC, Pati A (2015). Microbial species delineation using whole genome sequences. Nucleic Acids Res.

[9] Brynildsrud OB, Pepperell CS, Suffys P, Grandjean L, Monteserin J, Debech N, Bohlin J, Alfsnes K, Pettersson JO-H, Kirkeleite I, Fandinho F, da Silva MA, Perdigao J, Portugal I, Viveiros M, Clark T, Caws M, Dunstan S, Thai PVK, Lopez B, Ritacco V, Kitchen A, Brown TS, van Soolingen D, O’Neill MB, Holt KE, Feil EJ, Mathema B, Balloux F, Eldholm V. Global expansion of Mycobacterium tuberculosis lineage 4 shaped by colonial migration and local adaptation. Sci Adv. 2018; 4(10):eaat5869. Sourced from Microsoft Academic - https://academic.microsoft.com/paper/2898083195. 10.1126/sciadv.aat5869 PMC6192687 30345355

[10] Merker M, Blin C, Mona S, Duforet-Frebourg N, Lecher S, Willery E (2015). Evolutionary history and global spread of the Mycobacterium tuberculosis Beijing lineage. Nat Genet.

[11] Yin Q-q, Liu H-c, Jiao W-w, Li Q-j, Han R, Tian J-l, Liu Z-g, Zhao X-q, Li Y-j, Wan K-l, Shen A-d, Mokrousov I (2016). Evolutionary history and ongoing transmission of phylogenetic sublineages of Mycobacterium tuberculosis Beijing genotype in China. Sci Rep.

[12] Namouchi A, Didelot X, Schöck U, Gicquel B, Rocha EPC (2012). After the bottleneck: Genome-wide diversification of the mycobacterium tuberculosis complex by mutation, recombination, and natural selection. Genome Res.

[13] DeJesus MA, Gerrick ER, Xu W, Park SW, Long JE, Boutte CC, Rubin EJ, Schnappinger D, Ehrt S, Fortune SM, Sassetti CM, Ioerger TR, Stallings CL, Manoil C, Lampe D (2017). Comprehensive Essentiality Analysis of the *Mycobacterium tuberculosis* Genome via Saturating Transposon Mutagenesis. mBio.

[14] Bateman A, Martin M-J, Orchard S, Magrane M, Agivetova R, Ahmad S, Alpi E, Bowler-Barnett EH, Britto R, Bursteinas B, Bye-A-Jee H, Coetzee R, Cukura A, Silva AD, Denny P, Dogan T, Ebenezer T, Fan J, Castro LG, Garmiri P, Georghiou G, Gonzales L, Hatton-Ellis E, Hussein A, Ignatchenko A, Insana G, Ishtiaq R, Jokinen P, Joshi V, Jyothi D, Lock A, Lopez R, Luciani A, Luo J, Lussi Y, MacDougall A, Madeira F, Mahmoudy M, Menchi M, Mishra A, Moulang K, Nightingale A, Oliveira CS, Pundir S, Qi G, Raj S, Rice D, Lopez MR, Saidi R, Sampson J, Sawford T, Speretta E, Turner E, Tyagi N, Vasudev P, Volynkin V, Warner K, Watkins X, Zaru R, Zellner H, Bridge A, Poux S, Redaschi N, Aimo L, Argoud-Puy G, Auchincloss A, Axelsen K, Bansal P, Baratin D, Blatter M-C, Bolleman J, Boutet E, Breuza L, Casals-Casas C, de Castro E, Echioukh KC, Coudert E, Cuche B, Doche M, Dornevil D, Estreicher A, Famiglietti ML, Feuermann M, Gasteiger E, Gehant S, Gerritsen V, Gos A, Gruaz-Gumowski N, Hinz U, Hulo C, Hyka-Nouspikel N, Jungo F, Keller G, Kerhornou A, Lara V, Mercier PL, Lieberherr D, Lombardot T, Martin X, Masson P, Morgat A, Neto TB, Paesano S, Pedruzzi I, Pilbout S, Pourcel L, Pozzato M, Pruess M, Rivoire C, Sigrist C, Sonesson K, Stutz A, Sundaram S, Tognolli M, Verbregue L, Wu CH, Arighi CN, Arminski L, Chen C, Chen Y, Garavelli JS, Huang H, Laiho K, McGarvey P, Natale DA, Ross K, Vinayaka CR, Wang Q, Wang Y, Yeh L-S, Zhang J, Ruch P, Teodoro D (2021). UniProt: the universal protein knowledgebase in 2021. Nucleic Acids Res.

[15] Kapopoulou A, Lew JM, Cole ST (2011). The mycobrowser portal: a comprehensive and manually annotated resource for mycobacterial genomes. Tuberculosis.

[16] Brule CE, Grayhack EJ (2017). Synonymous codons: Choose wisely for expression. Trends Genet.

[17] Lin T-W, Melgar MM, Kurth DG, Swamidass SJ, Purdon J, Tseng T, Gago GM, Baldi P, Gramajo HC, Tsai SC (2006). Structure-based inhibitor design of accd5, an essential acyl-coa carboxylase carboxyltransferase domain of mycobacterium tuberculosis. Proc Natl Acad Sci U S A.

[18] Jackson M. The Mycobacterial Cell EnvelopeŮLipids. Cold Spring Harb Perspect Med. 2014; 4(10):a021105. Sourced from Microsoft Academic - https://academic.microsoft.com/paper/2152257820. 10.1101/cshperspect.a021105 PMC4200213 25104772

[19] Yang C, Luo T, Sun G, Qiao K, Sun G, DeRiemer K, Mei J, Gao Q (2012). Mycobacterium tuberculosis Beijing Strains Favor Transmission but Not Drug Resistance in China. Clin Infect Dis.

[20] Zhao L-L, Li M-C, Liu H-C, Xiao T-Y, Li G-L, Zhao X-Q, Liu Z-G, Wan K-L (2019). Beijing genotype of mycobacterium tuberculosis is less associated with drug resistance in South China. Int J Antimicrob Agents.

[21] Loutet MG, Davidson JA, Brown T, Dedicoat M, Thomas HL, Lalor MK (2018). Acquired resistance to antituberculosis drugs in England, Wales, and Northern Ireland, 2000?2015. Emerg Infect Dis.

[22] Meehan CJ, Goig GA, Kohl TA, Verboven L, Dippenaar A, Ezewudo M, Farhat MR, Guthrie JL, Laukens K, Miotto P, Ofori-Anyinam B, Dreyer V, Supply P, Suresh A, Utpatel C, van Soolingen D, Zhou Y, Ashton PM, Brites D, Cabibbe AM, de Jong BC, de Vos M, Menardo F, Gagneux S, Gao Q, Heupink TH, Liu Q, Loiseau C, Rigouts L, Rodwell TC, Tagliani E, Walker TM, Warren RM, Zhao Y, Zignol M, Schito M, Gardy J, Cirillo DM, Niemann S, Comas I, Rie AV (2019). Whole genome sequencing of mycobacterium tuberculosis: current standards and open issues. Nat Rev Microbiol.

[23] Faksri K, Xia E, Ong RT-H, Tan JH, Nonghanphithak D, Makhao N, Thamnongdee N, Thanormchat A, Phurattanakornkul A, Rattanarangsee S, Ratanajaraya C, Suriyaphol P, Prammananan T, Teo Y-Y, Chaiprasert A (2018). Comparative whole-genome sequence analysis of mycobacterium tuberculosis isolated from tuberculous meningitis and pulmonary tuberculosis patients. Sci Rep.

[24] Tantivitayakul P, Ruangchai W, Juthayothin T, Smittipat N, Disratthakit A, Mahasirimongkol S, Viratyosin W, Tokunaga K, Palittapongarnpim P (2020). Homoplastic single nucleotide polymorphisms contributed to phenotypic diversity in mycobacterium tuberculosis. Sci Rep.

[25] Gygli SM, Borrell S, Trauner A, Gagneux S (2017). Antimicrobial resistance in mycobacterium tuberculosis: mechanistic and evolutionary perspectives. FEMS Microbiol Rev.

[26] Bolger AM, Lohse M, Usadel B (2014). Trimmomatic: a flexible trimmer for illumina sequence data. Bioinformatics.

[27] Langmead B, Salzberg SL (2012). Fast gapped-read alignment with bowtie 2. Nat Methods.

[28] Koboldt DC, Zhang Q, Larson DE, Shen D, McLellan MD, Lin L, Miller CA, Mardis ER, Ding L, Wilson RK (2012). Varscan 2: Somatic mutation and copy number alteration discovery in cancer by exome sequencing. Genome Res.

[29] Li H, Handsaker B, Wysoker A, Fennell T, Ruan J, Homer N, Marth G, Abecasis G, Durbin R (2009). The sequence alignment/map format and samtools. Bioinformatics.

[30] Coll F, Preston M, Guerra-Assunção JA, Hill-Cawthorn G, Harris D, Perdigão J, Viveiros M, Portugal I, Drobniewski F, Gagneux S, Glynn JR, Pain A, Parkhill J, McNerney R, Martin N, Clark TG (2014). Polytb: a genomic variation map for mycobacterium tuberculosis. Tuberculosis.

[31] Chernyaeva E, Rotkevich M, Krasheninnikova K, Yurchenko A, Vyazovaya A, Mokrousov I, Solovieva N, Zhuravlev V, Yablonsky P, O’Brien SJ (2018). Whole-genome analysis of Mycobacterium tuberculosis from patients with tuberculous spondylitis, Russia. Emerg Infect Dis.

[32] Phelan JE, O’Sullivan DM, Machado D, Ramos J, Oppong YEA, Campino S, O’Grady J, McNerney R, Hibberd ML, Viveiros M, Huggett JF, Clark TG (2019). Integrating informatics tools and portable sequencing technology for rapid detection of resistance to anti-tuberculous drugs. Genome Med.

[33] Cook DE, Andersen EC (2017). VCF-kit: assorted utilities for the variant call format. Bioinformatics.

[34] Stamatakis A (2014). RAxML version 8: a tool for phylogenetic analysis and post-analysis of large phylogenies. Bioinformatics.

[35] Bankevich A, Nurk S, Antipov D, Gurevich AA, Dvorkin M, Kulikov AS, Lesin VM, Nikolenko SI, Pham S, Prjibelski AD, Pyshkin AV, Sirotkin AV, Vyahhi N, Tesler G, Alekseyev MA, Pevzner PA (2012). Spades: A new genome assembly algorithm and its applications to single-cell sequencing. J Comput Biol.

[36] Lin H-H, Liao Y-C (2016). Accurate binning of metagenomic contigs via automated clustering sequences using information of genomic signatures and marker genes. Sci Rep.

[37] Coll F, McNerney R, Guerra-Assunção JA, Glynn JR, Perdigão J, Viveiros M, Portugal I, Pain A, Martin N, Clark TG (2014). A robust SNP barcode for typing Mycobacterium tuberculosis complex strains. Nat Commun.

[38] Xia E, Teo Y-Y, Ong RT-H (2016). SpoTyping: fast and accurate in silico Mycobacterium spoligotyping from sequence reads. Genome Med.

[39] Letunic I, Bork P (2019). Interactive Tree Of Life (iTOL) v4: recent updates and new developments. Nucleic Acids Res.

